# Editorial: Recent advances in nanofabricated delivery systems of bioactive components for food applications

**DOI:** 10.3389/fnut.2022.1124487

**Published:** 2023-01-04

**Authors:** Kandi Sridhar, Minaxi Sharma

**Affiliations:** ^1^Department of Food Technology, Koneru Lakshmaiah Education Foundation Deemed to be University, Vaddeswaram, India; ^2^Haute Ecole Provinciale de Hainaut-Condorcet, Ath, Belgium

**Keywords:** nanotechnology, design and fabrication, encapsulation, delivery system, food science

Nanotechnology is a multidisciplinary and rapidly evolving field, in which materials at atomic, molecular, and macromolecular scales are manipulated with sizes around 1–100 nm. Over the years, nanotechnology has emerged in development of healthier, safer, high-quality functional foods, nano-nutraceuticals, and safe delivery of natural bioactive components at targeted sites. In this regard, nanotechnological approaches in the field of food science and technology enhanced the bioavailability of natural bioactive ingredients and provided long-term stability against environmental conditions. For example, nanofabricated delivery systems, such as nanoemulsions, nanoliposomes, polymeric/biopolymeric nanoparticles (NPs), solid lipid NPs, nanohydrogel beads, nanostructured lipid NPs, dendrimers in organic NPs, nanocomposites, and supercritical fluid-based or metal/metal oxide NPs are developed with various synthetic or natural polymers to improve the functional performance of natural bioactive compounds and ensuring delivery at the desired time and rate. Recently, many food processing industries showcased interest in the use of food nanostructured ingredients for sustainable development of consumer-oriented functional or nutraceutical foods with enhanced quality attributes.

In this context, to improve scientific knowledge and understanding on the application of nanotechnology in food processing, two experts (Dr. Kandi Sridhar and Dr. Minaxi Sharma) in micro/nano encapsulation, bioactive compounds, and food fortification proposed a riveting Research Topic entitled “Recent Advances in Nanofabricated Delivery Systems of Bioactive Components for Food Applications” to be published in Frontiers in Nutrition under the section Nutrition and Food Science Technology. This Research Topic covered the recent research innovations and developments in the nanoengineered delivery systems of food-bioactive compounds and their role in the sustainable development of functional or future foods toward nutritional security. Moreover, it covered in-depth engineered approaches, particularly in the design and fabrication of nano delivery systems for specific food applications. Researchers around the world were invited to contribute research and/or review articles that focus on (but are not limited to) nanotechnology-based systems for delivery of bioactive compounds; nanofabrication methods and nanostructures in food systems; behavior of various nanofabricated delivery systems in food models; safety and health implications of nanofabricated delivery systems; sustainable food production, quality, and bioactive compound delivery in food formulations; future perspectives in the design and fabrication of multiple nanoscale delivery systems.

This special Research Topic exclusively attracted the global researchers and received good quality submissions. Under this special Research Topic, a total of four articles (two research and two review) containing 34 highly reputed authors were published that included biopolymeric NPs and other nanoencapsulation methods for effective management of inflammation and cancer. The research articles exclusively focused on the nanoencapsulation of bioactive compounds and their potential applications, while review articles provided the overview on the nanofabricated delivery systems. A study by Pathak et al. reviewed the recent developments in biopolymeric NPs for the effective delivery of bioactive compounds for cancer therapy and concluded the significant advantages (i.e., high specificity, high stability, controlled drug release, and high drug–carrying capacity) of NPs in drug delivery systems. This review concluded the biopolymeric NPs as widely used anticancer bioactive delivery systems for cancer therapy in the pharmaceutical and medical areas. A captivating research led by Dr. Mithun Rudrapal, in collaboration with his group from Saudi Arabia, fabricated the silver NPs using embelin derived from *Embelia ribes* and studied its anticancer activity (Jagtap et al.). Also, a research group discussed several encapsulating techniques for probiotics, release kinetics, and challenges of nanoencapsulated bacteria in the food industry (Singh et al.). Another study developed *Woodfordia fruticosa* extract nanoemulsion and observed the Influence of processing treatments on the droplet size of nanoemulsion and its assessment for *in vitro* antimicrobial and anti-inflammatory activities. They concluded that the nanoemulsion developed from *Woodfordia fruticosa* extract can be used as a potential antimicrobial agent in food applications (Najda et al.).

In summary, the findings of above-mentioned research and reviews addressed the application of nanotechnology in food, particularly, showed the nanoencapsulation as an effective targeted delivery system to enhance the bioavailability of natural extracts, embelin, and other bioactive compounds. These outstanding research and review articles are open access publications (©2022 by authors) distributed under the terms and conditions of the Creative Commons Attribution (CC BY) license. Conversely more research should be needed to establish the therapeutic efficiency and target delivery of bioactive compounds. Overall, the published articles in this topic contribute to existing knowledge of nanofabricated delivery systems for development of healthy, sustainable, and consumer-oriented functional foods to feed the growing population.

As a note, we are extremely delighted to organize and disseminate this Research Topic to a wider audience through Frontiers in Nutrition. We strongly believe that this Research Topic attracted the academic and non-academic communities, and stakeholders, and improved the readership of the journal, Frontiers in Nutrition. We further believe that this Research Topic will be the basis for further in-depth research into nano-based delivery systems.

## Author contributions

KS: conceptualization, data curation, methodology, formal analysis, software, writing—original draft, validation, and visualization. MS: conceptualization, data curation, validation, writing—review and editing, supervision, visualization, and validation. Both authors contributed to the article and approved the submitted version.

